# Low polymorphisms in *pfact*, *pfugt* and *pfcarl* genes in African *Plasmodium falciparum* isolates and absence of association with susceptibility to common anti-malarial drugs

**DOI:** 10.1186/s12936-019-2919-3

**Published:** 2019-08-28

**Authors:** Francis Tsombeng Foguim, Marie Gladys Robert, Mamadou Wagué Gueye, Mathieu Gendrot, Silman Diawara, Joel Mosnier, Rémy Amalvict, Nicolas Benoit, Raymond Bercion, Bécaye Fall, Marylin Madamet, Bruno Pradines, The French National Reference Centre for Imported Malaria Study Group, V. Augis, D. Basset, P. Bastien, F. Benoit-Vical, A. Berry, P. Brouqui, M. Cividin, P. Delaunay, L. Delhaes, M. Drancourt, T. Gaillard, A. Genin, E. Garnotel, E. Javelle, C. L’Ollivier, M. Leveque, D. Malvy, P. Marty, M. Mechain, G. Ménard, P. Millet, P. Minodier, A. Mottard, P. Parola, R. Piarroux, C. Pomares-Estran, M.-C. Receveur, A. Robin, E. Sappa, H. Savini, F. Simon, Y. Sterkers, C. Surcouf, E. Varlet, A. Wolff

**Affiliations:** 1 Unité Parasitologie et Entomologie, Département de Microbiologie et de maladies infectieuses, Institut de recherche biomédicale des armées, IHU Méditerranée Infection, 19-21 Boulevard Jean Moulin, 13005 Marseille, France; 2 IRD, SSA, AP-HM, VITROME, Aix Marseille Université, Marseille, France; 3 IHU Méditerranée Infection, Marseille, France; 4 Fédération des laboratoires, Hôpital Principal de Dakar, Dakar, Senegal; 5 Centre national de référence du Paludisme, Marseille, France; 6 Laboratoire d’analyses médicales, Institut Pasteur de Dakar, Dakar, Senegal

**Keywords:** Malaria, *Plasmodium falciparum*, Anti-malarial drug, In vitro, Resistance, Molecular marker, PfACT, PfUGT, PfCARL

## Abstract

**Background:**

Resistance to all available anti-malarial drugs has emerged and spread including artemisinin derivatives and their partner drugs. Several genes involved in artemisinin and partner drugs resistance, such as *pfcrt*, *pfmdr1*, *pfK13* or *pfpm2*, have been identified. However, these genes do not properly explain anti-malarial drug resistance, and more particularly clinical failures observed in Africa. Mutations in genes encoding for *Plasmodium falciparum* proteins, such as *P. falciparum* Acetyl-CoA transporter (PfACT), *P. falciparum* UDP-galactose transporter (PfUGT) and *P. falciparum* cyclic amine resistance locus (PfCARL) have recently been associated to resistance to imidazolopiperazines and other unrelated drugs.

**Methods:**

Mutations on *pfugt*, *pfact* and *pfcarl* were characterized on 86 isolates collected in Dakar, Senegal and 173 samples collected from patients hospitalized in France after a travel in African countries from 2015 and 2016 to assess their potential association with ex vivo susceptibility to chloroquine, quinine, lumefantrine, monodesethylamodiaquine, mefloquine, dihydroartemisinin, artesunate, doxycycline, pyronaridine and piperaquine.

**Results:**

No mutations were found on the genes *pfugt* and *pfact*. None of the *pfcarl* described mutations were identified in these samples from Africa. The K784N mutation was found in one sample and the K734M mutation was identified on 7.9% of all samples for *pfcarl.* The only significant differences in ex vivo susceptibility according to the K734M mutation were observed for pyronaridine for African isolates from imported malaria and for doxycycline for Senegalese parasites.

**Conclusion:**

No evidence was found of involvement of these genes in reduced susceptibility to standard anti-malarial drugs in African *P. falciparum* isolates.

## Background

According to the World Health Organization (WHO) recommendations, endemic countries have adopted the use of artemisinin-based combination therapy (ACT) to treat uncomplicated malaria cases [1]. Despite considerable progress, 219 million new malaria cases were reported with 435,000 deaths in 2017 [2]. Currently, artemisinin-based combinations are the most potent available anti-malarial drugs that are used for the reduction of the malaria global burden. Combination of a long acting drug with the short acting artemisinin is used to provide a protection against emergence of resistant parasites [3]. Mefloquine, lumefantrine, amodiaquine, and more recently piperaquine and pyronaridine are the available artemisinin-based partner drugs in ACT. Resistance to artemisinin described as a delayed parasite clearance after treatment has emerged in Southeast Asia [4, 5]. It was also reported that low treatment success rate of ACT was associated with resistance to the partner drugs. This resistance has been described in Southeast Asia and may occur in Africa soon [6, 7].

In 2013, a molecular marker strongly associated with artemisinin resistance was identified as mutations in the Kelch 13 propeller domain (*pfk13*) in the Southeast Asia, but none of these mutations are yet documented in Africa [8, 9]. *Pfk13* and/or the Ring-stage Survival Assay (RSA) are now used as tools to track artemisinin and artemisinin derivatives resistance in endemic areas in addition with epidemiological survey. However, recent studies proved that *pfk13* is not the only marker to be associated with artemisinin resistance [10]. Clinical failures with ACT have also been observed in African patients with *P. falciparum* parasites without *pfk13* polymorphism [11–15]. Polymorphisms in other genes, like *P. falciparum* actin-binding protein coronin, *P. falciparum* ubiquitin carboxyl-terminal hydrolase 1 (*pfubp1*) or *P. falciparum* clathrin vesicle-associated adaptor 2 µ subunit (*pfap2mu*), have been also found to be associated with artemisinin resistance in African isolates [16, 17].

Additionally, resistance has also emerged to dihydroartemisinin–piperaquine, the most recently marketed ACT, in Cambodia and Vietnam [18–21]. In vitro and in vivo resistance to piperaquine has been associated with amplification of copy number of the *plasmepsin II* gene (*pfpm2*) in Cambodian isolates [22, 23]. However, amplification of this gene seems to be not associated with piperaquine in vitro and in vivo resistance particularly in Africa [24–29]. In conclusion, predictive molecular markers to track resistance to ACT in Africa are not yet identified.

Drug efficacy is modulated by parasite membrane proteins that are involved in drug transport. Two parasites membrane proteins, the *Plasmodium falciparum* chloroquine resistance transporter (PfCRT) and the *P. falciparum* multidrug resistance protein 1 (PfMDR1), both localized on the membrane of the digestive food vacuole, have been involved in drug resistance [30–32]. These proteins play an important role in trafficking of drugs between the parasite cytosol and the food vacuole. Their association with quinoline resistance has been demonstrated in many studies [30, 33–35].

But other less studied proteins may be involved in molecules traffic within the parasite. The *P. falciparum* Acetyl-CoA transporter (PfACT) and the *P. falciparum* UDP-galactose transporter (PfUGT) [36] are examples of major facilitator superfamily transporters and may share similar function [37]. The protein PfACT function is not known yet, but its parasite localization and its homologues form in other organisms suggest that this protein may be involved in intracellular translocation of small molecules including metabolites, nucleosides, oligosaccharides, amino-acids, oxyanions and drugs. These two putative transporters have been associated with in vitro resistance to imidazolopiperazines, and more particularly to KAF156 and GNF179, two new potential antimalarial compounds that are under clinical evaluation [38]. KAF156 showed high in vitro activity and in vivo efficacy against *P. falciparum* and *P. vivax* and in vitro and in vivo transmission blocking activity [39, 40]. KAF156 did not show in vitro cross-resistance with artemisinin and lumefantrine [38]. GNF179 was active in vitro against blood stages as well as liver stages [41]. Resistant parasites to KAF156 and GNF179 generated in vitro showed different mutations (A94T, R108K, S110R, D165N, C183*, S242*, L253* and G559K) in the *pfact* gene and a substitution of a phenylalanine by a valine at the position 37 of the gene *pfugt* (F37V) [35]. Additionally, the generation of resistant parasites to KAF156 and GNF179 lead to mutations (L830V, S1076N/I, V1103L, I1139K) in the *P. falciparum* cyclic amine resistance locus (PfCARL) [38, 39, 41–43]. *Pfcarl* plays a role in protein folding within the endoplasmic reticulum [44]. Mutations in *pfcarl* did not lead to in vitro resistance to artemisinin, chloroquine and mefloquine in two mutant strains [42]. These three genes seem to be multidrug-resistance genes specific to resistance to benzimidazolyl piperidines and imidazolopiperazines [38, 42, 43]. However, the data on cross-resistance with standard anti-malarial drugs were obtained from in vitro selection of *P. falciparum* mutant clones. Neither the involvement of these three genes in resistance to imidazolopiperazines nor cross-resistance with standard anti-malarial drugs have been assessed in field isolates. There are no data on polymorphisms and their prevalence in natural parasite populations, or in the involvement of these three genes on the susceptibility of ACT partner drugs such as piperaquine, pyronaridine, lumefantrine or amodiaquine against field *P. falciparum* isolates.

The present study aimed to evaluate the prevalence of polymorphisms in *pfact*, *pfugt* and *pfcarl* genes and to evaluate their association with reduced susceptibility to common anti-malarial drugs on 259 *P. falciparum* African isolates.

## Methods

### Sample collection

**Fig. 1:**
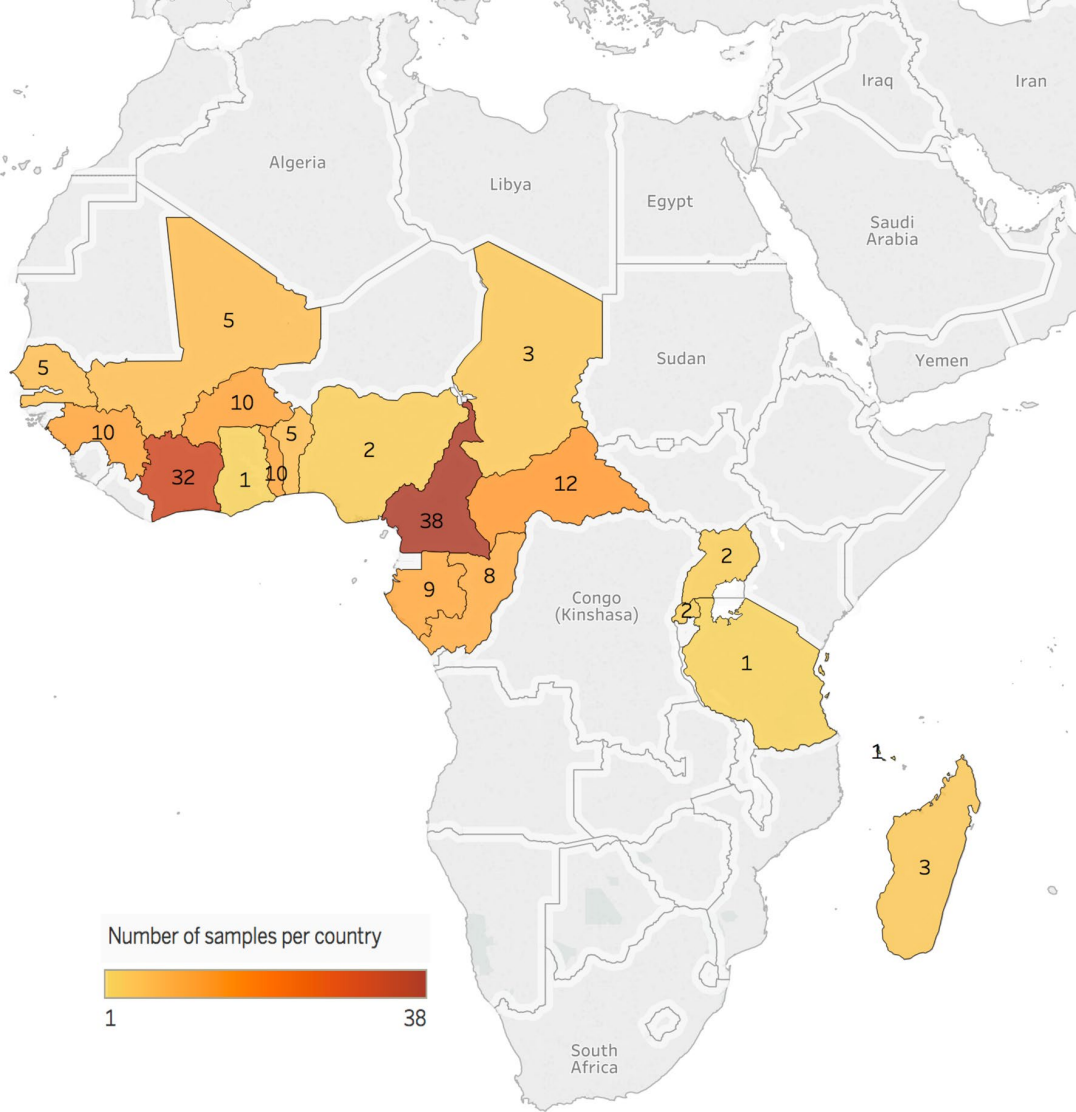
Geographical repartition and isolate number per country of the *Plasmodium falciparum* isolates collected between 2015 and 2016 from patients hospitalized in France with imported malaria from Africa

Eighty-six of the samples used were collected from falciparum malaria patients, who were recruited at the Hôpital Principal de Dakar, Senegal after the rainy seasons between 2013 and 2015 in the context of studies on evaluation of anti-malarial drug resistance [15, 45–47]. A total of 173 samples collected between 2015 and 2016 from patients hospitalized in France with imported malaria from a malaria-endemic country, especially from Cameroon, Côte d’Ivoire, Central African Republic, Burkina Faso, Togo, Gabon, Guinea and Senegal (Fig. 1) were additionally used to complete the study. Twelve samples have an unknown African origin. The samples were sent from different civilian or military hospitals of the French National Reference Centre for Imported Malaria network (Aix en Provence, Bordeaux, Fréjus, Marseille, Montpellier, Nice, Toulon and Toulouse) to the French National Reference Centre for Malaria (IRBA, IHU Méditerranée Infection Marseille).

Peripheral venous blood samples were collected in Vacutainer^®^ ACD tubes (Becton–Dickinson, Rutherford, NJ, USA) prior to patient treatment for parasite detection. The diagnosis was performed on thin blood smears that were stained using a RAL^®^ kit (Réactifs RAL, Paris, France), based on eosin and methylene blue, to determine the *P. falciparum* density. The diagnosis of *P. falciparum* mono-infection was confirmed by real time PCR (LightCycler 2.0, Roche Group, Switzerland), as previously described [48]. An aliquot of each sample was collected and stored at − 20 °C for molecular study. Parasitized erythrocytes were washed three times in RPMI 1640 medium (Invitrogen, Paisley, UK) buffered with 25 mM HEPES and 25 mM NaHCO_3_. If parasitaemia exceeded 0.1%, infected erythrocytes were diluted to 0.1% with uninfected erythrocytes (human blood type A+) and resuspended in RPMI 1640 medium supplemented with 10% human serum (Abcys S.A. Paris, France), for a final haematocrit of 1.5%. The susceptibility of the isolates to the different anti-malarial drugs was assessed without culture adaptation.

### Drugs and ex vivo assay

Chloroquine (CQ), quinine (QN), dihydroartemisinin (DHA) and doxycycline (DOX) were obtained from Sigma (Saint Louis, MO, USA), monodesethylamodiaquine (DQ) from the World Health Organization (Geneva, Switzerland), mefloquine (MQ) from Roche (Paris, France), lumefantrine (LMF) from Novartis Pharma (Basel, Switzerland), and artesunate (AS), piperaquine (PPQ) and pyronaridine (PND) from Shin Poong Pharm Co. (Seoul, Korea).

For each experiment, parasitized erythrocytes (final parasitemia at 0.5% and a final haematocrit at 1.5%) were aliquoted into 96-well plates that were pre-dosed with a concentration gradient of anti-malarial drugs. The plates were incubated for 72 h at 37 °C in controlled atmosphere at 85% N_2_, 10% O_2_, 5% CO_2_ for imported isolates and in a sealed bag with atmospheric generators for capnophilic bacteria using Genbag CO2^®^ at 5% CO_2_ and 15% O_2_ (BioMérieux, Marcy l’Etoile, France) for Senegalese isolates [49]. The drug susceptibility assay was performed using the HRP2 ELISA-based assay Malaria Ag Celisa kit (ref KM2159, Cellabs PTY LDT, Brookvale, Australia), as previously described [46].

Each batch of plates was validated using the CQ-resistant W2 strain (isolated in Indochina; obtained from MR4, VA, USA) in four independent experiments using the same conditions described below.

The mean 50% inhibitory concentration (IC_50_) values for the chloroquine-resistant W2 strain for the different batches used over 2 years in controlled atmosphere at 85% N_2_, 10% O_2_, 5% CO_2_ were 484 ± 40 nM for CQ, 388 ± 29 nM for QN, 97 ± 18 nM for DQ, 1.0 ± 0.4 nM for LMF, 26.3 ± 3.1 nM for MQ, 54.1 ± 5.4 nM for PPQ, 20.4 ± 3.4 nM for PND, 2.5 ± 0.4 nM for DHA, 1.5 ± 0.3 nM for AS and 11.5 ± 1.9 µM for DOX. A comparison of the W2 susceptibility data of the ten anti-malarial drugs between the different batches of plates indicated that there was no significant difference in the responses to anti-malarial drugs over the 2 years (0.583 < p < 0.993). The cut-off values for the reduced ex vivo susceptibility or resistance were as follows: 100 nM (CQ), 800 nM (QN), 80 nM (DQ), 30 nM (MQ), 150 nM (LMF), 135 nM (PPQ), 60 nM (PND), 10.5 nM (DHA and AS) and 35 µM (DOX) [50, 51].

The mean IC_50_ values for the W2 strain for the different batches used during the 3 years using atmospheric generators for capnophilic bacteria were 292 nM for CQ, 275 nM for QN, 72 nM for DQ, 13.7 nM for LMF, 15.4 nM for MQ, 32.5 nM for PPQ, 26.4 nM for PND, 1.27 nM for DHA, and 10.7 µM for DOX. A comparison of W2 susceptibility data for the nine anti-malarial drugs indicated that there was no significant difference in the responses to anti-malarial drugs over the 3 years (0.39 < p < 0.95). The cut-off values for the reduced ex vivo susceptibility or resistance were as follows: 77 nM (CQ), 611 nM (QN), 61 nM (DQ), 30 nM (MQ), 115 nM (LMF), 135 nM (PPQ), 60 nM (PND), 12 nM (DHA and AS) and 37 µM (DOX) [46, 47, 52].

The polymorphic genetic markers *msp1* and *msp2* and microsatellite markers specific to *P. falciparum* were genotyped at least once a month to verify W2 clonality [53, 54].

### Nucleic acid extraction

Total genomic DNA of each sample was isolated and purified using the QIAamp^®^ DNA Mini kit according to the manufacturer’s recommendations (Qiagen, Hilden, Germany).

### Genotyping of *pfact*, *pfugt* and *pfcarl*

The three genes, *pfact* (PF3D7_1036800), *pfugt* (PF3D7_1113300) and *pfcarl* (PF3D7_0321900), were amplified by polymerase chain reaction using the oligonucleotide primer pairs described in Table 1.

**Table 1:** Forward and reverse primers, hybridization temperature (Tm) and MgCl_2_ concentration used for PCR

Gene	Forward and reverse primers	Tm
*pfugt* (PF3D7_1113300)	Pfugt-F 5′-GCT CAG GTA TGT TTT GGA AG-3′ Pfugt-R 5′-GTC CAG TAA GTC CGT CAC AT-3′	52 °C
*pfact* (PF3D7_1036800)	Pfact-1F 5′-TTG TGT AAC CCC CAC TAA AC-3′	54 °C
	Pfact-1R 5′-TTA TCG TCA CAC TTT TGT GC-3′ Pfact_seq 1F 5′-CTA TTT TGC AGT TTT ACG ATG-3′	54 °C
	Pfact-2F 5′-TGA TTA CAC TGA TAA GGA ATT TTG-3′ Pfact-2R 5′-TTC GTT CTC CAA TCT TCT AAA-3′	48 °C
*pfcarl* (PF3D7_0321900)	Pfcarl-F 5′-TTG CCA TGA TTT GAA GTA CA-3′ Pfcarl-R 5′-AAC CAT TTT CGT ATT CAT GTT-3′	50 °C

Two primer pairs were used to amplify the *pfact* fragments (1042 and 407 nucleotides). The reaction mixture contained 200 ng of genomic DNA, 0.32 µM of each primer, 1× final of reaction buffer (750 mM of Tris–HCl, 200 mM of (NH_4_)_2_SO_4_, 0.1% (v/v) Tween 20 and stabilizer, pH 8.8), 2.5 mM of MgCl_2_, 200 µM of dNTP mixture and 1 U of Hot Diamond Taq^®^ polymerase (Eurogentec, Liège, Belgium) in a final volume of 25 µL. The thermal cycler (Life Eco V 2.04; Bioer, China) was programmed as follows: 95 °C for 10 min, 40 cycles of 95 °C for 30 s, hybridization temperature for 45 s (Table 1), 72 °C for 1 min 20 s, and a final 10-min extension step at 72 °C.

A fragment of 600 nucleotides of *pfugt* gene was amplified using the two primer pairs described in Table 1. The reaction mixture contained 200 ng of genomic DNA, 0.32 µM of each primer, 1× final of reaction buffer (750 mM of Tris–HCl, 200 mM of (NH_4_)_2_SO_4_, 0.1% (v/v) Tween 20 and stabilizer, pH 8.8), 2.5 mM of MgCl_2_, 200 µM of dNTP mixture and 1 U of Hot Diamond Taq^®^ polymerase (Eurogentec, Liège, Belgium) in a final volume of 25 µL. The thermal cycler (Life Eco V 2.04; Bioer, China) was programmed as follows: 95 °C for 10 min, 40 cycles of 95 °C for 30 s, hybridization temperature for 45 s (Table 1), 72 °C for 45 s, and a final 10-min extension step at 72 °C.

To analyse *pfcarl* mutations, a fragment of 821 nucleotides was amplified using the specific primer pair described in Table 1. The reaction mixture contained 200 ng of genomic DNA, 0.32 µM of each primer, 1× final of reaction buffer (750 mM of Tris–HCl, 200 mM of (NH_4_)_2_SO_4_, 0.1% (v/v) Tween 20 and stabilizer, pH 8.8), 2.5 mM of MgCl_2_, 200 µM of dNTP mixture and 1 U of Hot Diamond Taq^®^ polymerase (Eurogentec, Liège, Belgium) in a final volume of 25 µL. The thermal cycler (Life Eco V 2.04; Bioer, China) was programmed as follows: 95 °C for 10 min, 40 cycles of 95 °C for 30 s, hybridization temperature for 45 s (Table 1), 72 °C for 1 min, and a final 10-min extension step at 72 °C.

The purified amplicons were sequenced using corresponding PCR primers and a sequencing primer for *pfact* first fragment (Table 1) on an ABI Prism 3100 analyser (Applied Biosystems, Villebon sur Yvette, France) according to the manufacturers’ instructions. Sequences were aligned and compared with the corresponding sequences of the *P. falciparum* 3D7 using Vector NTI 10.3.0 (Invitrogen, Cergy Pontoise, France) to identify potential SNPs.

### Data and statistical analysis

Samples and genotype distribution were performed on Tableau Desktop (Version 10.3.2). Plots of IC_50_ distribution were performed using R software. Statistical analyses were performed on SPSS, Version 16 (IBM, USA). Normally distributed IC_50_s data for each drug were assessed by the Kolmogorov–Smirnov test.

## Results

### Ex vivo susceptibility to anti-malarial drugs

**Fig. 2:**
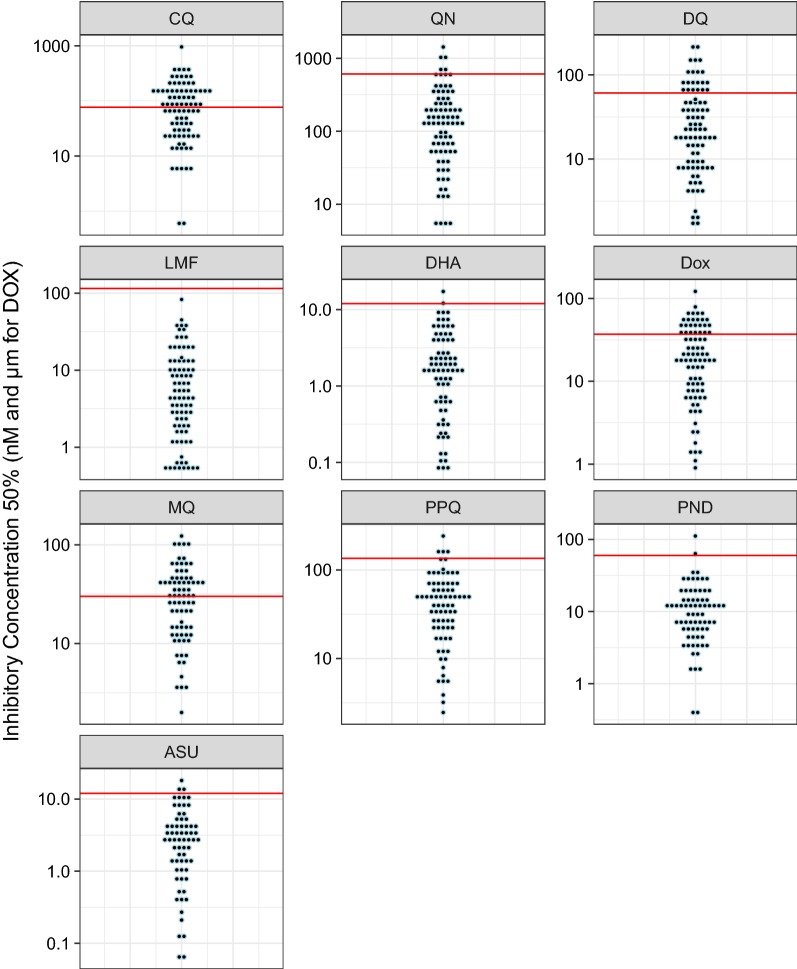
Dot plot of the IC_50_ total distribution of each Senegalese *Plasmodium falciparum* isolate assessed ex vivo for chloroquine (CQ), quinine (QN), monodesethylamodiaquine (DQ), mefloquine (MQ), lumefantrine (LMF), piperaquine (PPQ), pyronaridine (PND), dihydroartemisinin (DHA), artesunate (AS) and doxycycline (DOX). The red line represents the in vitro threshold of reduced susceptibility

**Fig. 3:**
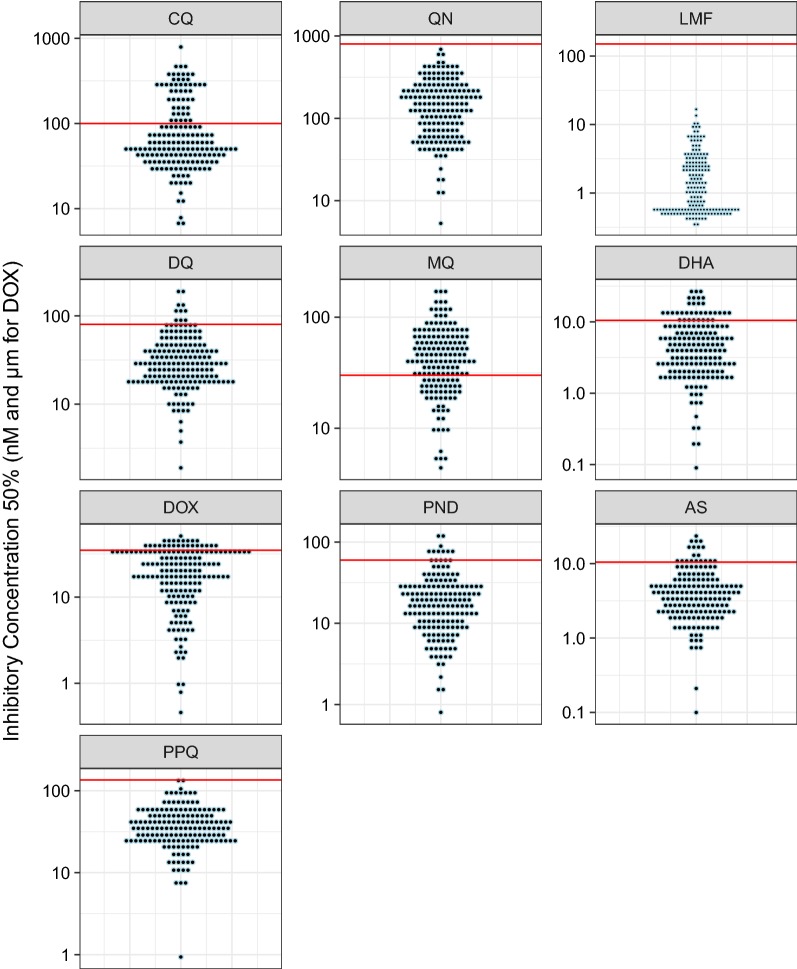
Dot plot of the IC_50_ total distribution of each *Plasmodium falciparum* isolate from imported malaria to France assessed ex vivo for chloroquine (CQ), quinine (QN), monodesethylamodiaquine (DQ), mefloquine (MQ), lumefantrine (LMF), piperaquine (PPQ), pyronaridine (PND), dihydroartemisinin (DHA), artesunate (AS) and doxycycline (DOX). The red line represents the in vitro threshold of reduced susceptibility

The average parameters of the IC_50_ values for the ten anti-malarial drugs are presented in Table 2. The distribution of the IC_50_ values are showed in Fig. 2 for the Senegalese isolates and in Fig. 3 for malaria imported isolates.

**Table 2:** Average parameters of *P. falciparum* susceptibility to chloroquine (CQ), quinine (QN), monodesethylamodiaquine (DQ), mefloquine (MQ), lumefantrine (LMF), pyronaridine (PND), piperaquine (PPQ), dihydroartemisinin (DHA), artesunate (AS) and doxycycline (DOX)

Drugs	Isolates from Senegal (n = 86)				African isolates from imported malaria analyzed for *pfcarl* (n = 173)			
	IC_50_		Geometric mean	Resistance %	IC_50_		Geometric mean	Resistance %
	Min	Max			Min	Max		
CQ	0.6	954.9	60.0	48.6	6.27	791.6	69.7	28.3
QN	5	1429.8	113	5.7	5.29	690.1	127.8	0
DQ	1.6	227.3	21.7	23.6	1.9	196.43	29.2	8.1
MQ	2	109.2	24.8	48.5	4.4	173.43	39.5	67.7
LMF	0.5	82.9	4.53	0	0.33	16.66	1.4	0
PND	0.4	116.6	16.4	3.1	0.8	122.96	16.5	6.1
PPQ	3.2	241.9	37.4	6.1	0.94	137.51	34.3	0.6
DHA	0.08	17.28	1.36	1.5	0.09	28.11	4.1	17.5
AS	0.06	18.06	2.18	3.5	0.1	23.56	3.7	9.8
DOX	0.9	121.6	23.0	28.2	0.46	41.14	16.2	17.3

IC_50_ in nM for CQ, QN, DQ, MQ, LMF, PND, PPQ, DHA, AS

IC_50_ in µM for DOX

### Gene sequence polymorphism analysis

The previously identified mutations at position 94, 108, 110, 165, 183, 242, 253 and 559 in the *pfact* gene were not detected and no other polymorphism was identified in the 259 African isolates. For the gene *pfugt*, all samples were also wild type at position 37 and no other polymorphism was identified within the sequences.

None of the mutations of the *pfcarl* gene involved in imidazolopiperazine resistance was found in African *P. falciparum* isolates. Three new mutations were detected: the K784N mutation present in one isolate, the K734M mutation (8.9% on Senegalese samples and 7.5% on malaria imported samples) and the K903E mutation which was found on both all Senegalese and malaria imported valid sequences (100%). The only significant differences in ex vivo susceptibility according to the K734M mutation were observed for PND for African isolates from imported malaria (p = 0.028; 22.1 nM vs. 39.2 nM) and for DOX for Senegalese parasites (p = 0.034; 26.4 µM vs. 8.0 µM) (Table 3). The difference in PND IC_50_s according to the wild type/mutant haplotype of PfCARL was not significant by pooling all the IC_50_s from imported and Senegalese isolates (20.4 nM vs. 30.4 nM; p = 0.202). There was no significant difference between the prevalences of 734 M mutated parasites in susceptible isolates and that in parasites with reduced susceptibility to the different anti-malarial drugs tested in the present study (p values between 0.053 and 1 [Fisher’s exact test]) (Table 4).

**Table 3:** Ex vivo susceptibility of African *Plasmodium falciparum* isolates to chloroquine (CQ), quinine (QN), monodesethylamodiaquine (DQ), mefloquine (MQ), lumefantrine (LMF), piperaquine (PPQ), pyronaridine (PND), dihydroartemisinin (DHA), artesunate (AS) and doxycycline (DOX) according to the K734M mutation in the *pfcarl* gene

Drug	Isolates from Senegal					African isolates from imported malaria				
	Wild-type K734 (n = 61)		Mutated 734M (n = 6)		p-value	Wild-type K734 (n = 160)		Mutated 734 M (n = 13)		p-value
	Mean IC_50_	Min and Max	Mean IC_50_	Min and Max		Mean IC_50_	Min and Max	Mean IC_50_	Min and Max	
CQ	110.7	0.6/954.9	87.7	17.0/163	0.835	109.1	7.2/791.6	107.6	6.27/383.8	0.616
QN	225.8	5/1429.8	69.0	15.0/140.1	0.087	176.0	11.53/690.1	137.8	5.29/338.3	0.227
DQ	36.9	1.6/202.6	41.8	2.4/140.0	0.690	37.1	3.72/196.4	39.1	1.9/105.6	0.588
MQ	37.6	2.0/123.0	19.6	10.0/44.0	0.088	48.8	4.4/173.4	60.0	21.16/172.6	0.398
LMF	9.9	0.5/82.9	5.9	1.2/12.6	0.970	2.2	0.33/13.5	2.6	0.37/16.7	0.704
PPQ	56.1	3.2/241.9	37.9	12.0/100.0	0.350	39.9	0.94/137.5	47.4	14.3/96.8	0.293
PND	15.4	0.4/111.6	9.5	4.8/15.2	0.700	22.1	0.8/114.6	39.2	7.09/122.9	0.028
DHA	2.9	0.8/17.3	2.1	0.09/9.2	0.101	6.0	0.19/28.1	7.1	0.09/23.3	0.404
AS	4.2	0.06/18.1	4.6	2.5/8.5	0.709	5.0	0.1/23.6	5.8	1.9/17.9	0.362
DOX	26.4	0.9/79.0	8.0	1.5/18.4	0.034	22.0	0.79/51.1	16.3	0.46/39.4	0.144

**Table 4:** Prevalences of *Plasmodium falciparum* isolates with the 734M mutation in the *P. falciparum* cyclic amine resistance locus (*pfcarl*) according to parasite susceptibility to chloroquine (CQ), quinine (QN), lumefantrine (LMF), desethylamodiaquine (DQ), mefloquine (MQ), pyronaridine, (PND), piperaquine (PPQ), dihydroartemisinin (DHA), artesunate (AS) and doxycycline (DOX)

Drug	Reduced-susceptible cutoff	% of isolates with the 734M mutation (no. of isolates with the 734M mutation/total no. of susceptible or resistant isolates		p value (Fisher’s exact test)
		Susceptible parasites	Parasites with reduced susceptibility	
CQ	77^a^ or 100^b^ nM	7.7 (12/155)	8.2 (7/85)	1
QN	611^a^ or 800^b^ nM	8.0 (19/235)	0 (0/5)	1
LMF	150 nM	8.0 (19/235)	0 (0/0)	1
DQ	61^a^ or 80^b^ nM	7.2 (15/209)	12.9 (4/31)	0.28
MQ	30 nM	7.4 (7/95)	6.9 (10/145)	1
PND	60 nM	6.9 (16/233)	28.6 (2/7)	0.089
PPQ	135 nM	7.2 (17/236)	0 (0/4)	1
DHA	12^a^ or 10.5^b^ nM	7.4 (16/217)	13.0 (3/23)	0.41
AS	12 or 10.5 nM	6.7 (15/225)	25.0 (3/12)	0.053
DOX	37^a^ or 35^b^ nM	8.2 (16/194)	6.5 (3/46)	1

^a^Cutoff estimated for ex vivo test in seal bag with atmospheric generator using Genbag CO2^®^ [52]

^b^Cutoff estimated for ex vivo test in controlled atmosphere for imported isolates [50, 51]

## Discussion

The aim of the present study was to determine whether described SNPs in the genes *pfact*, *pfugt* and *pfcarl*, involved in imidazolopiperazine resistance, are found in African isolates, their prevalence and if these mutations are associated with common anti-malarial drug susceptibility. The main limitation of this study is the low number of parasites with reduced susceptibility to LMF (0% for Senegalese isolates and imported isolates), QN (5.7% for Senegalese isolates and 0% for imported isolates), PND (3.1% for Senegalese isolates and 6.1% for imported isolates) and PPQ (6.1% for Senegalese isolates and 0.6% for imported isolates). However, IC_50_ values were distributed in a broad way (Figs. 2, 3). There was no polymorphism in the analysed sequence of *pfact* and *pfugt*. None of the mutations of the *pfcarl* gene involved in imidazolopiperazine resistance was found in African *P. falciparum* isolates but three other ones were identified: the K784N mutation present in one isolate, the K734M mutation (7.9%) (prevalence of 8.9% in Senegalese samples and 7.5% in malaria imported samples from Africa) and the K903E mutation (100%). These mutations were also found in *P. falciparum* sequences filed on PlasmoDB in similar proportions: 0.4% for K784N, 11% for K734M and 99% for K903E mutation. The K734M seemed to be not associated with susceptibility to standard anti-malarial drugs. No evidence was found of prevalence difference between susceptible isolates and parasites with reduced susceptibility. Only parasites collected from African imported malaria carrying the K734M mutation were significantly less susceptible to pyronaridine than wild *P. falciparum* parasites (39.2 nM vs. 22.1 nM; p = 0.028). However, these data should be taken with caution due to the low number of samples, and more specially parasites with reduced susceptibility to anti-malarial drugs. It is necessary to further assess more *P. falciparum* isolates to ascertain the potential association between the *pfcarl* K734M mutation and reduced susceptibility to pyronaridine.

A limitation of this kind of study is the strength of the correlation between ex vivo or in vitro studies and therapeutic efficacy assays. Clinical failures with dihydroartemisinin/piperaquine in Cambodia were associated with resistant phenotype but this association has not been shown yet in Africa [22, 23, 55]. Association between these two methods is not fully established for some anti-malarial drugs like mefloquine, lumefantrine, piperaquine, pyronaridine, and more particularly in Africa. The main explanations for a lack of correlation are that in vitro assays and clinical studies of therapeutic efficacy do not address the same biological and clinical endpoints and the cut off for in vitro reduced susceptibility are usually fixed arbitrarily without any reference to predictable clinical and parasitological response [56]. Many factors, and more specially host factors like acquired immunity, nutritional status, pharmacokinetic characteristics, interact in drug in vivo efficacy. However, the major criteria for a valid in vitro or ex vivo threshold should be the association with clinical outcome.

Additionally, in the absence of standardized ex vivo and in vitro tests, it is very difficult to compare data from different laboratories. IC_50_ and cut-off values for in vitro resistance are specific to the methodology. The in vitro effects and the IC_50_ values for anti-malarial drugs depend on incubation conditions [57, 58], gas conditions (e.g., the effects of O_2_ and CO_2_) [45, 59], and methodology (e.g., use of an isotopic test vs. an immune-enzymatic test) [60]. These differences in methodology must be taken into account when comparing and analysing resistance data from different studies. The use of a reference strain as internal control is essential to validate and compare data obtained with several batches of plates.

## Conclusion

None of the mutations of the *pfact*, *pfugt* and *pfcarl* genes involved in imidazolopiperazine or benzimidazolyl piperidine resistance was found in 259 African *P. falciparum* isolates. The prevalence of these mutations in Africa was very low. This absence of mutations involved in imidazolopiperazine or benzimidazolyl piperidine resistance suggests that the *pfact*, *pfugt* and *pfcarl* genes are not involved in quinoline ex vivo resistance (28.3 to 48.6% of resistance to chloroquine, 8.1 to 23.6% of resistance to desethylamodiaquine or 48.5 to 67.7% of resistance to mefloquine) and in doxycycline reduced susceptibility (17.3 to 28.2%) in the 259 *P. falciparum* African isolates which were evaluated in the present study. Additionally, the 734M mutation identified in the *pfcarl* gene at a rate of 7.9% was not associated with ex vivo susceptibility to standard anti-malarial drugs. This absence of identification of the mutations in *pfact*, *pfugt* and *pfcarl* genes, which are involved in imidazolopiperazine resistance, in 259 African *P. falciparum* isolates, is suggesting a very low prevalence of resistant parasites, encouraging data for the use of KAF156 and GNF179 for malaria treatment.

## Data Availability

The datasets analysed in this study are available from the corresponding author on reasonable request.

## Abbreviations

ACT: artemisinin-based combination therapy
AS: artesunate
CQ: chloroquine
DHA: dihydroartemisinin
DNA: deoxyribonucleic acid
DQ: monodesethylamodiaquine
DOX: doxycycline
IC_50_: 50% inhibitory concentration
LMF: lumefantrine
MQ: mefloquine
PfACT: *Plasmodium falciparum* Acetyl-CoA transporter
*pfap2mu*: *P. falciparum* clathrin vesicle-associated adaptor 2 µ subunit
PfCARL: *Plasmodium falciparum* cyclic amine resistance locus
PfCRT: *Plasmodium falciparum* chloroquine resistance transporter
PfK13: *Plasmodium falciparum* Kelch 13
PfMDR1: *Plasmodium falciparum* multidrug resistance 1 gene
*pfubp1*: *P. falciparum* ubiquitin carboxyl-terminal hydrolase 1 gene
PfUGT: *Plasmodium falciparum* UDP-galactose transporter
PND: pyronaridine
PPQ: piperaquine
QN: quinine
SNP: single nucleotide polymorphism
WHO: World Health Organization
